# Alternative Treatment Options to ALK Inhibitor Monotherapy for EML4-ALK-Driven Lung Cancer

**DOI:** 10.3390/cancers14143452

**Published:** 2022-07-15

**Authors:** Savvas Papageorgiou, Sarah L. Pashley, Laura O’Regan, Sam Khan, Richard Bayliss, Andrew M. Fry

**Affiliations:** 1 Department of Molecular and Cell Biology, University of Leicester, Lancaster Road, Leicester LE1 7RH, UK; sp807@le.ac.uk (S.P.); slp66@le.ac.uk (S.L.P.); lor2@le.ac.uk (L.O.); 2 Leicester Cancer Research Centre (LCRC), Department of Genetics and Genome Biology, University of Leicester, Robert Kilpatrick Clinical Sciences Building, Leicester LE2 7LX, UK; sk504@le.ac.uk; 3 Astbury Centre for Structural Molecular Biology, Faculty of Biological Sciences, University of Leeds, Leeds LS2 9JT, UK; r.w.bayliss@leeds.ac.uk

**Keywords:** EML4-ALK, NSCLC, ALK inhibitors, TKIs, chemotherapy, radiotherapy, immunotherapy, microtubule poisons

## Abstract

**Simple Summary:**

Lung cancer remains one of the most common and difficult cancers to treat. However, excellent progress in identifying the genetic causes of lung cancers has revealed many of the mutant proteins driving progression of this cancer. This in turn has led to development of new precision medicines that target these proteins providing significant improvement to patient outcomes. Unfortunately, this benefit tends to be relatively short-lived as the cancers develop resistance to these targeted agents. In this review, we look specifically at cancers driven by the EML4-ALK protein that is present in around 5% of lung cancer patients, occurring more frequently in young non-smokers. We consider the recent evidence for how resistance develops to current clinically approved targeted ALK inhibitors in these patients. Furthermore, we explore how additional research is revealing exciting alternative treatment options that may lead to a more sustained response and increased long-term survival for these patients.

**Abstract:**

EML4-ALK is an oncogenic fusion protein that accounts for approximately 5% of NSCLC cases. Targeted inhibitors of ALK are the standard of care treatment, often leading to a good initial response. Sadly, some patients do not respond well, and most will develop resistance over time, emphasizing the need for alternative treatments. This review discusses recent advances in our understanding of the mechanisms behind EML4-ALK-driven NSCLC progression and the opportunities they present for alternative treatment options to ALK inhibitor monotherapy. Targeting ALK-dependent signalling pathways can overcome resistance that has developed due to mutations in the ALK catalytic domain, as well as through activation of bypass mechanisms that utilise the same pathways. We also consider evidence for polytherapy approaches that combine targeted inhibition of these pathways with ALK inhibitors. Lastly, we review combination approaches that use targeted inhibitors of ALK together with chemotherapy, radiotherapy or immunotherapy. Throughout this article, we highlight the importance of alternative breakpoints in the *EML4* gene that result in the generation of distinct EML4-ALK variants with different biological and pathological properties and consider monotherapy and polytherapy approaches that may be selective to particular variants.

## 1. Introduction

### 1.1. Epidemiology and Genetics of Lung Cancer

Lung cancer is the leading type of cancer in males and among the top three for females around the globe. Regardless of gender, it is also the leading cause of cancer-related mortality worldwide [1]. Lung cancer is classified as either small cell lung cancer (~15%) (SCLC) or non-small cell lung cancer (~85%) (NSCLC), with the latter further categorized into adenocarcinomas, squamous cell or large cell carcinomas [2]. Lung cancer is strongly associated with tobacco smoking and the introduction of tobacco control programmes has resulted in a sharp decline in both incidence and mortality [3,4,5]. Despite such programmes, smoking continues to be a key risk factor, along with other environmental risk factors (e.g., ionizing radiation, air pollution), and age and genetic risk factors. Notably, less than 10% of lung cancer patients survive for 10 years or more [6,7].

Several genetic changes have been identified as the main drivers of NSCLC, with *EGFR* and *KRAS* mutations being ranked as the most common followed by *ALK* rearrangements [8]. The discovery of oncogenic driver mutations resulted in the development of targeted therapies that have revolutionised the treatment paradigm for NSCLC, resulting in better long-term patient outcomes [9]. Interestingly, NSCLC patients with either an *EGFR* mutation or *ALK* rearrangement are more likely to be non- or light-smokers, suggesting that the proportion of these will increase as smoking-related changes reduce [10]. A recent review by the National Institute for Health, identified three molecular subtypes of cancer in non-smokers: piano, a slow growing subtype associated with progenitor cells with few mutations, mezzo-forte, harbouring EGFR-related mutations which grew quicker than the piano group, and forte, which had genomic changes similar to those who smoked, demonstrating that there is heterogeneity in the pathogenesis of tumours in this population of patients. An increased focus on never smoker NSCLC patients will hopefully in time lead to further personalised therapy options for these individuals, who typically present later compared to their smoking counterparts often due to the vague nature of their symptoms [11].

### 1.2. ALK Rearrangements

The *anaplastic lymphoma kinase* (*ALK*) gene was initially discovered in 1994 in non-Hodgkin’s lymphoma, as a result of a chromosomal translocation involving the *nucleophosmin (NPM)* gene that led to the formation of the NPM-ALK oncogenic fusion protein [12]. *ALK* is situated on the short arm of chromosome 2 and encodes a transmembrane receptor tyrosine kinase (RTK). It is highly expressed in neuronal cells during the early stages of cellular development, but expression is almost completely absent in mature cells [13]. Since 1994, *ALK* rearrangements involving a variety of genes have been reported in several types of malignancies, including colorectal, breast and NSCLC [14,15]. Most prominently, in-frame fusion of *ALK* with the *EML4* gene, which encodes the echinoderm microtubule (MT)-associated protein (EMAP)-like 4 (EML) protein to create the EML4-ALK fusion protein, is found in approximately 5% of NSCLC cases [10].

### 1.3. The EML4-ALK Oncogenic Fusion

The *EML4* gene is the most common fusion partner of *ALK* and EML4-ALK was first identified in 2007 in ~7% of Japanese NSCLC patients [16,17]. The EML4-ALK fusion is now reported among ~4–6% of NSCLC patients and has allowed for the development of personalized therapies for ALK+ NSCLC patients [18,19]. A number of diagnostic approaches are currently in use for identification of the EML4-ALK fusion in ALK+ patients. Practice regarding molecular diagnostics will vary widely driven by different health care systems and availability of testing and treatment options resulting in heterogeneity of approaches. In addition, biopsy sample size may be limited and must be handled with care, ensuring tissue is used sparingly. Guidelines vary globally, but the cornerstone of diagnosis is based on fluorescence in situ hybridization (FISH), immunohistochemistry (IHC) or more recently next-generation sequencing (NGS). The United States Food and Drug Administration (FDA) has a list of approved testing techniques as a companion diagnostic for each ALK inhibitor therapy. Testing platforms currently include IHC, FISH and NGS [20]. In Europe, historically the standard of care for detection of an ALK rearrangement is FISH, though in some countries IHC can also be used in conjunction with another approach for confirmation [21,22,23,24,25].

**Figure 1:**
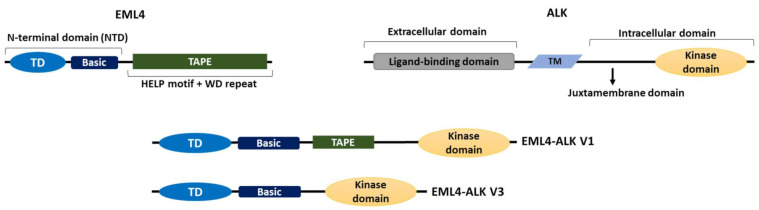
The domain organizations of EML4, ALK and EML4-ALK V1 and V3. A cartoon illustrating the domain organizations of EML4, ALK and the two most commonly occurring EML4-ALK variants, the long V1 (E13; A20) and the short V3 (E6; A20); “E” and “A” represent the exons of EML4 and ALK, respectively, that are fused in each variant. Both variants contain the intracellular kinase domain of ALK and the TD and basic region of EML4. V1 also expresses a truncated TAPE domain from EML4 whereas V3 completely lacks the TAPE domain. TM = transmembrane domain, TD = trimerization domain.

The EML4-ALK oncogenic fusion results from a paracentric inversion on the short arm of chromosome 2 that joins the regions coding for the N-terminus of *EML4* to that encoding the C-terminal kinase domain of *ALK* [16]. Different breakpoints in the *EML4* gene give rise to distinct EML4-ALK variants (Figure 1) [16,26]. To date, more than 15 variants have been discovered in NSCLC, some of which have multiple isoforms as a result of alternative splicing. However, variants 1 (V1) and 3 (V3) are the most common by some margin, and together represent approximately 80% of EML4-ALK cases [16,26,27]. Routinely variant testing is not assessed clinically in most healthcare systems globally.

There are six human EML proteins, named EML1 to EML6. These are homologues of the echinoderm microtubule-associated protein (EMAP), which was isolated from unfertilised sea urchin eggs in 1993 [28]. EML1 to EML4 have a similar organization with an N-terminal domain (NTD) made up of a coiled-coil followed by a region rich in basic residues. Structural evidence indicates that the coiled-coil leads to trimer assembly, hence this motif has been called a trimerization domain (TD). Localization studies demonstrate that the TD together with the basic region is essential for microtubule binding of these EMLs [29,30]. Meanwhile, a series of tryptophan-aspartate (WD) repeats fold into two seven-bladed β-propellers that, along with a hydrophobic EML-like protein (HELP) motif, form the C-terminal tandem atypical propeller in EMLs (TAPE) domain of EML1 to EML4 [30,31]. EML5 and EML6 are somewhat different in organization, lacking the NTD and having three contiguous repeats of the TAPE domain encoded in a single polypeptide [31]. Therefore, consistent with the importance of the NTD for microtubule binding, the lack of NTD in EML5 and EML6 suggests that they may be unable to bind microtubules, but this remains to be tested.

The ALK protein comprises an extracellular domain required for ligand binding, a transmembrane sequence, and an intracellular domain, which contains the tyrosine kinase [32]. All EML4-ALK variants contain the complete ALK tyrosine kinase domain but lack the transmembrane and extracellular regions. Importantly though, they differ in the amount of the EML4 protein they include, due to distinct breakpoints in the *EML4* gene. For example, V1, V2 and V4 possess the EML4 NTD and part of the TAPE domain and are known as the long variants, while V3 and V5 have some or all of the NTD, but none of the TAPE domain and are referred to as the short variants [30] (Figure 1). The presence of the TAPE domain in the long EML4-ALK variants significantly reduces the stability of the fusion proteins, and deletion of different parts of the TAPE domain from the long variants resulted in a stability profile similar to that of the short variant, V3, in which the TAPE domain is completely absent [33].

Considerable research has focused on elucidating the mechanisms behind *EML4-ALK* fusion-driven cancer progression, largely due to the fact that they are the most common *ALK* rearrangement in NSCLC. Firstly, given that all variants contain the EML4 TD, it is widely assumed that trimerization of EML4-ALK monomers via the TD facilitates the trans-autophosphorylation of *ALK*, which gives rise to a constitutively active tyrosine kinase [16,29,34]. However, in other respects there are several important differences in the biological properties of the long and short variants.

EML4-ALK V1 and V3 are the two most common variants, and are representative of the long and short variants, respectively. Although both variants contain the TD and basic region of EML4, only V3 localizes to microtubules in a similar manner to the wild-type EML4 protein, while V1 is strictly cytoplasmic, potentially because the incomplete TAPE domain sequence perturbs binding to microtubules [29]. As previously mentioned, the presence of the partial TAPE domain within the long EML4-ALK variants reduces their stability and they require the heat-shock protein 90 (HSP90) chaperone to maintain their expression. Hence, it is also possible that association with HSP90 perturbs interaction of the long variants with microtubules [35]. However, recent evidence suggests that the presence of the 12N blade region within the TAPE domain in the long variants inhibits their microtubule localization, because deletion of this region results in V1 associating with microtubules [36]. These differences in localization and stability could explain the profound variability in both prognosis and response to targeted treatment for patients with different variants. For example, cells expressing the short variant V3, exhibit a much lower sensitivity to the ALK tyrosine kinase inhibitor (TKI), crizotinib, than those expressing the long variant V2 [33,35]. Moreover, Christopoulos et al. (2018) found that patients with V3 exhibited enhanced metastasis and reduced overall survival (OS) compared to those with V1 or V2, supporting the hypothesis that EML4-ALK V3, and potentially short variants more broadly, are more aggressive variants, resulting in poorer patient outcomes [37].

Intriguingly, the oligomerization and activation of both EML4-ALK V1 and V3 enables the formation of cytoplasmic compartments, described as granules or foci, that contain downstream components of several ALK-dependent signalling pathways, including those of the RAS/MAPK and JAK/STAT pathways, along with the EML4-ALK protein [36,38,39]. Both ceritinib and lorlatinib, two ALK inhibitors, can dissolve these cytoplasmic foci and redirect V3, but not V1, to microtubules, which mirrors the localization of a catalytically inactive V3 mutant. However, this does not rule out the possibility that active V3 also localizes to microtubules as well as within cytoplasmic foci. Nonetheless, the formation of these cytoplasmic granules appears to be dependent on ALK activity. Furthermore, constitutively active ALK mutants stabilized these foci even in the presence of ALK inhibitors [36]. Interestingly, the disruption of the EML4 portion of the fusion protein prevents the formation of cytoplasmic granules, highlighting the potential importance of EML4 in forming these condensates [39].

### 1.4. Targeted ALK Inhibitors and Resistance Mechanisms

Targeted ALK inhibitors have become the standard of care treatment for ALK positive (ALK+) NSCLC patients [40,41]. A growing number of ALK TKIs have been approved for use, including first- (crizotinib; [18,19,42,43]), second- [alectinib, ceritinib, brigatinib; [44,45,46,47,48] and third-generation (lorlatinib, ensartinib, entrectinib; [49]) inhibitors with each consecutive generation having enhanced clinical properties such as blood-brain-barrier penetration. However, despite their initial efficacy, patients often become resistant to ALK TKIs. It is thus imperative to understand the mechanisms behind this resistance and develop alternative approaches to treatment [19,43,50]. Furthermore, fourth-generation inhibitors such as TPX-0131 are also in pre-clinical studies, and preliminary data has indicated that TPX-0131 has high potency against both wild-type ALK and ALK that is resistant to previous generation ALK inhibitors [51].

To date, several different ALK mutations have been identified that promote resistance to ALK inhibitors, with the majority sitting within the ATP-binding pocket of the ALK catalytic domain. Interestingly, these can confer differential sensitivity to distinct ALK TKIs [52]. For instance, the ALK F1174V missense mutation confers resistance to ceritinib but sensitivity to alectinib, whereas conversely the ALK I1171S mutation leads to resistance to alectinib but sensitivity to ceritinib [53,54]. Meanwhile, an ALK F1245C mutation promotes resistance to the first-generation ALK inhibitor, crizotinib, while G1202R is the most common mutation observed in tumours resistant to second generation ALK TKIs [52,55]. Hence, characterization of the ALK sequence following development of resistance to one ALK TKI would facilitate selection of the most suitable subsequent treatment.

Other than *ALK* catalytic site mutations, additional resistance mechanisms have been described. For example, *ALK* amplification is also able to promote crizotinib resistance [56,57]. Furthermore, tumours can switch to *ALK*-independent growth through activation of “bypass” signalling pathways for growth and survival. Some of the most common bypass pathways involve EGFR, MAPK or IGF-IR [57,58,59]. Finally, the co-occurrence of EML4-ALK with other genetic changes—for example, TP53 mutation—can serve as a resistance mechanism by promoting cell survival and other tumour-related adaptations such as upregulation of *MYC*. Indeed, overexpression of *MYC,* a transcriptional regulator of multiple cancer-related processes, has been linked to resistance to crizotinib and alectinib [60,61].

### 1.5. Aim of Review

There have been many excellent review articles that summarise the oncogenic signalling pathways activated in NSCLC by the EML4-ALK fusion proteins as well as the mechanisms of action of ALK inhibitors and acquired resistance to these inhibitors e.g., [10,30,62,63]. By contrast, less has been published to date on potential alternatives to ALK TKIs or how ALK inhibitors could be used in combination with other treatment modalities, such as chemotherapy, radiotherapy, or immunotherapy, for the treatment of ALK+ NSCLC patients. The purpose of this review is to address this gap by providing a summary of recent advances in our understanding of the mechanisms underpinning EML4-ALK-driven NSCLC that might reveal alternative treatment options to ALK TKI monotherapy.

## 2. Targeting ALK-Dependent Signalling Pathways

**Figure 2:**
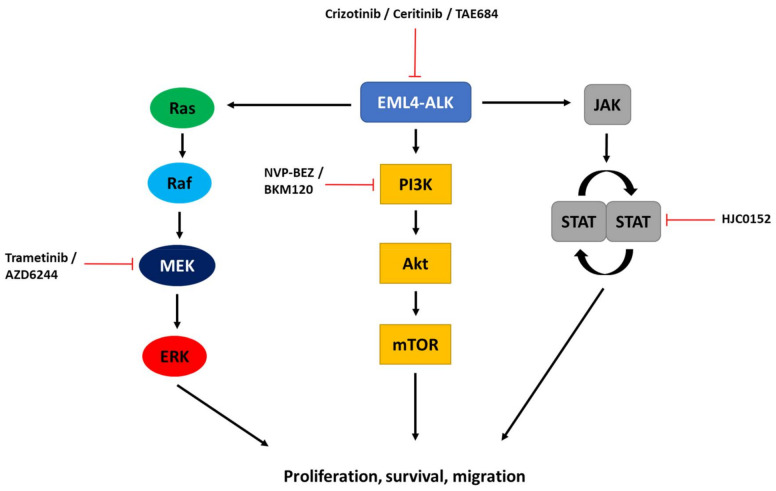
ALK-dependent signalling pathways in EML4-ALK-driven NSCLC. This schematic figure illustrates the three most prominent ALK-dependent signalling pathways activated by EML4-ALK: the MAPK, PI3K/AKT and JAK/STAT signalling pathways. The position at which selected pharmacological inhibitors (trametinib, AZD6244, NVP-BEZ, BKM120, HJC0152) that could act in combination with ALK inhibitors (crizotinib, ceritinib, TAE684) as discussed in the text is indicated. Combinations would work to inhibit activation of these pathways and suppress cancer cell proliferation, survival and migration.

Where it is found that resistance has arisen through mutations in the ALK catalytic domain, the most obvious alternative approach to ALK TKI monotherapy would be to target the ALK-dependent signalling pathways that occur downstream of ALK activation. The current evidence suggests that ALK signalling operates through three well-characterised pathways: the JAK/STAT, RAS/MAPK, and PI3K/AKT pathways (Figure 2). The Janus Kinase/Signal Transducers and Activators of the Transcription (JAK/STAT) pathway regulates a plethora of cancer-related processes, including proliferation, differentiation, migration, apoptosis, and survival [64]. Specifically, in vitro studies suggest that EML4-ALK V3 interacts with and activates the JAK2/STAT signalling pathway, while siRNA depletion of STAT6 leads to an increased rate of apoptosis of patient-derived cells expressing V3 [65]. Meanwhile, Shen et al. (2022) have shown that EML4-ALK V3 G1202R mutation can promote ceritinib resistance through activation of epithelial-to-mesenchymal transition (EMT) via STAT3 upregulation and consequent expression of its downstream target, the Slug transcription factor. A combination of HJC0152, a STAT3 inhibitor, and ceritinib was able to reverse the EMT phenotype and promote apoptosis in drug-resistant EML4-ALK V3 cells [55]. Hence, the JAK/STAT pathway appears particularly relevant to cells expressing EML4-ALK V3.

Signalling through the RAS/MAPK pathway is also activated in ALK+ NSCLC cells. Treatment of the V3 patient-derived cell line, H2228, with a combination of an experimental ALK TKI (TAE684) and AZD6244, a MEK inhibitor, blocked both the STAT3 and MAPK pathways. This led to a simultaneous increase in pro-apoptotic BIM and decrease in anti-apoptotic survivin expression [66]. Similarly, Hrustanovic et al. (2015) found that trametinib, a MEK inhibitor, plus crizotinib, caused enhanced apoptosis and diminished cell growth in the V1 patient-derived cell line, H3122, compared to either treatment alone both in vitro and in vivo [58]. Furthermore, they demonstrated that MAPK signalling is regulated by EML4-ALK through activation of all three RAS isoforms (i.e., H-, N- and K-RAS) and that their simultaneous knock-down led to a marked reduction in activation of MAPK signalling. Interestingly, they suggested that the EML4 HELP domain contributes to RAS activation, as deletion of this domain prevented MAPK activation [58].

The PI3K/AKT pathway is another important ALK-dependent signalling pathway [30]. In fact, co-occurrence of EML4-ALK V3 with an activating mutation within the PI3K signalling pathway was observed to elicit resistance to multiple ALK inhibitors [67]. Combined inhibition of PI3K and MAPK pathways was attempted in a murine model of EML4-ALK NSCLC using the PI3K and MEK inhibitors, NVP-BEZ and AZD6244, respectively. Despite impressive results regarding loss of cell viability in established NSCLC cell lines (i.e., H3122 cells which express V1), the combination only conferred modest benefit in an in vivo model. However, a polytherapy approach that combined the PI3K and MEK inhibitors with an ALK TKI was not attempted [68]. Yang et al. (2014) proposed that upregulation of PI3K acts as a resistance mechanism to ALK TKIs in EML4-ALK V3 patient-derived cells, and that PI3K inhibition restores sensitivity to those inhibitors. Furthermore, the combined use of TAE684 and BKM120, a PI3K inhibitor, produced synergistic effects in vitro through induction of apoptosis and perturbation of tumour growth [69].

An alternative approach to using inhibitors of the three signalling pathways mentioned above would be to target other less well studied mediators of ALK signalling. An et al. (2016) reported that the Crk-like (CRKL) adaptor protein is a mediator of ALK signalling in EML4-ALK NSCLC cells. They demonstrated that CRKL knock-down led to attenuated ALK signalling and reduced cell viability and migration [70]. Given that CRKL inhibitors are not currently available, one could indirectly inhibit CRKL by targeting upstream proteins, such as the ABL kinase, which has been shown to recruit CRKL [71,72]. Intriguingly, dasatinib, an ABL inhibitor, almost completely suppressed the phosphorylation and recruitment of CRKL and yielded a synergistic response when combined with crizotinib, suggesting that this combination may prevent or overcome resistance [70].

Of course, these signalling pathways can also be turned on by other receptors to bypass the requirement for ALK. For example, Tanizaki et al. (2012) reported not only an increase in the activation of three additional RTKs (EGFR, HER2, HER3), but also an increase in secretion of their ligand, epidermal growth factor (EGF), in EML4-ALK V1 patient-derived cells following administration of the ALK TKI, TAE684. Furthermore, they demonstrated that this led to activation of both the MAPK and JAK/STAT pathways, suggesting that the switch to EGF/EGFR/HER-dependent signalling results in TAE684 resistance. Indeed, treatment with an EGFR inhibitor induced apoptosis in these TAE684-resistant EML4-ALK V1 cells [73]. In addition, activation of EGFR in EML4-ALK V3 cells following administration of another ALK inhibitor, alectinib, was reported by Tani et al. (2016), who found that alectinib-resistant V3 cells expressed higher levels of TGFα, a ligand for EGFR [74]. In this case, combination with an EGFR inhibitor restored sensitivity to alectinib and promoted tumour regression in alectinib-resistant mouse models suggesting that resistance was mediated not by ALK mutations, but rather by upregulation of bypass pathways [74]. Several studies have reported activation of paracrine receptors, including EGFR and ErbB4, as another mode of resistance to ALK TKIs [75,76].

Interestingly, in one case, EML4-ALK expression served as a resistance mechanism to treatment of EGFR-mutant NSCLC with the EGFR inhibitor, osimertinib. Here, EGFR-dependent signalling switched to ALK-dependent activation, leading to acquired resistance to EGFR inhibition. This resistance was reversed when a combination of osimertinib and the ALK inhibitor, alectinib, was administered [77]. With this in mind, combination targeting of these signalling pathways may be effective both in ALK+ patients in which these bypass pathways are activated, and in patients for which the primary oncogenic driver is the more common EGFR mutation.

## 3. Targeting Pathways Independent of ALK Catalytic Activity

It is clear that cells expressing distinct variants of the EML4-ALK fusion oncogene have different sensitivities to ALK inhibitors [78,79,80,81]. Most notably, cells expressing V3 are generally more resistant to the ALK inhibitors developed so far than cells expressing other common variants, such as V1 [79,82,83,84]. For example, the 2-year progression-free survival (PFS) rate amongst patients treated with the first-generation inhibitor, crizotinib, was 76% for those harbouring V1 versus ~27% for those harbouring V3. When considering crizotinib-, alectinib- and ceritinib-treated patients, the 2-year PFS rates remain relatively unchanged; patients harbouring V3 have a much lower PFS rate than those with the V1 protein (~33% versus 69%, respectively) [84]. Given that all variants share the same ALK catalytic domain, this suggests that the EML4 portion of the fusion must be partly responsible for this observed resistance to ALK TKIs [30]. Furthermore, it is possible that different isoforms of the same variant might have an impact on ALK TKI sensitivity. Indeed, the V3b isoform was found to be more sensitive to ALK TKIs (crizotinib or TAE684) than the V3a isoform [33]. In fact, this study found that V3b exhibited a similar level of growth inhibition in response to these ALK TKIs as V1. Another study showed that most tumours with V3 harbour both V3a and V3b RNA isoforms, and that treatment with ALK TKIs increases the proportion of V3a relative to V3b over time [85]. This suggests that targeting the splicing machinery could be used as an adjuvant approach to ALK inhibition, to either prevent or stimulate expression of more or less resistant isoforms of EML4-ALK variants, respectively [82].

**Figure 3:**
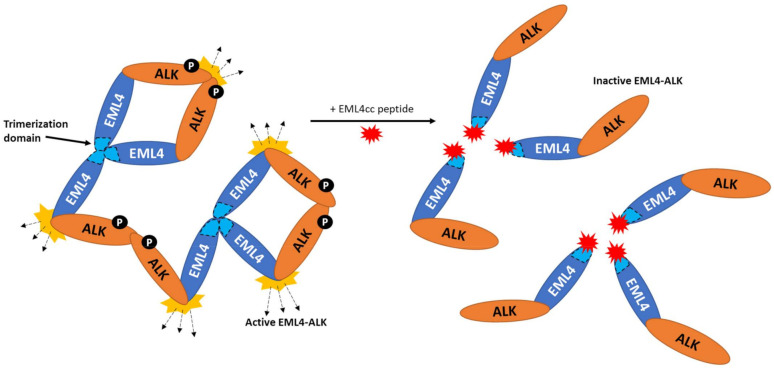
Blocking EML4-ALK trimerization. The illustration on the left shows how oligomerization via the trimerization domain of EML4 (dotted areas, coloured light blue) and the phosphorylation (P) of the ALK tyrosine kinase domain leads to ALK activation and downstream signalling. On the right, a synthetic peptide (EML4cc) analogous to the trimerization domain coiled-coil sequence (red star) blocks EML4-ALK trimerization through competitive TD binding and is shown to prevent activation of EML4-ALK.

As indicated earlier, it is assumed that TD-induced ALK oligomerization underlies the constitutive activation of the EML4-ALK fusion by enabling trans-autophosphorylation. Hirai et al. (2020) used competitive peptides to test the hypothesis that disrupting ALK oligomerization would result in reduced ALK activation. For this, they introduced synthetic peptides analogous to the EML4 TD coiled-coil sequence into cells to prevent trimerization of EML4-ALK (Figure 3). The peptides significantly reduced the growth of EML4-ALK V1 patient-derived cells and also diminished the activation of ALK and ALK-dependent signalling pathways. Intriguingly, a combination of these peptides with alectinib conferred a greater reduction in growth of V1 patient-derived cells compared to ALK TKI monotherapy [86]. However, stability, membrane permeability and the pharmacodynamic properties of the peptide need to be considered when considering their use in vivo [87,88,89,90]. Although this is still an approach aimed at preventing ALK kinase activation, it does represent an alternative approach to targeting the catalytic site and so is unlikely to lead to resistance through catalytic domain mutations.

The “short” EML4-ALK variants that lack any part of the TAPE domain are more stable than the “long” variants that contain a truncated portion of the TAPE domain [33]. The presence of the TAPE domain in the long variants disrupts the folding of the fusion protein forcing them to adapt a more open conformation and this provides access for HSP90 to bind exposed hydrophobic residues stabilizing the protein [33,35]. Hence, studies have tested the efficacy of HSP90 inhibitors in cancer cells with the longer variants of EML4-ALK. The combination of an ALK TKI with HSP90 inhibition not only yielded synergism in Ba/F3 cells expressing EML4-ALK V1, but also induced apoptosis in vitro and tumour regression in vivo of patient-derived cells expressing V1 [33,91]. Furthermore, Gower et al. (2016) demonstrated that resistance to first and second generation ALK TKIs could be reversed in vitro following administration of HSP90 inhibitors [92]. Despite these encouraging results in preclinical models, the use of HSP90 inhibitors in clinical trials for ALK+ NSCLC has so far met with disappointing results, with no improvement in outcomes compared to ALK TKI monotherapy alone [93,94].

Long non-coding RNAs (lncRNAs) regulate many pathological and physiological processes, including lung cancer [95,96]. Different lncRNAs have been identified in ALK TKI-resistant EML4-ALK-driven NSCLC cell lines that could be targeted to potentially reverse ALK inhibitor resistance [97,98]. For instance, Zhang et al. (2021) identified upregulation of Lnc01001 in crizotinib-resistant cells. Moreover, as Lnc01001 was associated with the IGF2BP2/MYC axis, inhibition of IGF2BP2 was found to repress crizotinib resistance and IGF2BP2 knock-down further reduced crizotinib resistance [98].

Loss of cell cycle or transcriptional control is also likely to contribute to acquired resistance to ALK TKIs. For example, upregulation of a variety of cyclin-dependent kinases (e.g., CDK1, CDK7 and CDK9) occurs upon treatment with crizotinib or alectinib. Selective inhibition of CDK9 with alvocidib or dinaciclib, and CDK7 and CDK12 with THZ1, led to induction of apoptosis in patient-derived cells expressing V1 or V3 through downregulation of anti-apoptotic genes, such as survivin or MYC [99]. CDK7 and CDK9 are involved in transcriptional regulation and, more specifically, in the binding of RNA polymerase II (Pol II) to gene promoters, and release and elongation steps, respectively [100]. Consistent with this, Paliouras et al. (2020) found that the CDK7/12 inhibitor prevented the binding of Pol II to the transcription start site, while the CDK9 inhibitor prevented release of Pol II from the transcription start site [99]. Hence, this study proposes the use of CDK inhibitors as an alternative to ALK TKI monotherapy following disease progression.

EML4 not only binds to microtubules via its NTD but also promotes microtubule stabilization [29,101]. As mentioned above, the short but not long EML4-ALK variants also associate with microtubules. A novel pathway has been described by O’Regan et al. (2020), in which cells expressing the short, but not long, variants form extended cytoplasmic protrusions and exhibit increased microtubule stability and enhanced migration [95]. These phenotypes are consistent with, and may be responsible for, the enhanced metastatic potential of tumours expressing EML4-ALK V3. Importantly, expression of an EML4-ALK V3 mutant that lacked ALK catalytic activity induced the same phenotypes, while treatment with crizotinib did not reverse these phenotypes in cells expressing wild-type V3. This would suggest that these consequences are not a result of ALK activity and thus are not prevented by ALK inhibitors.

**Figure 4:**
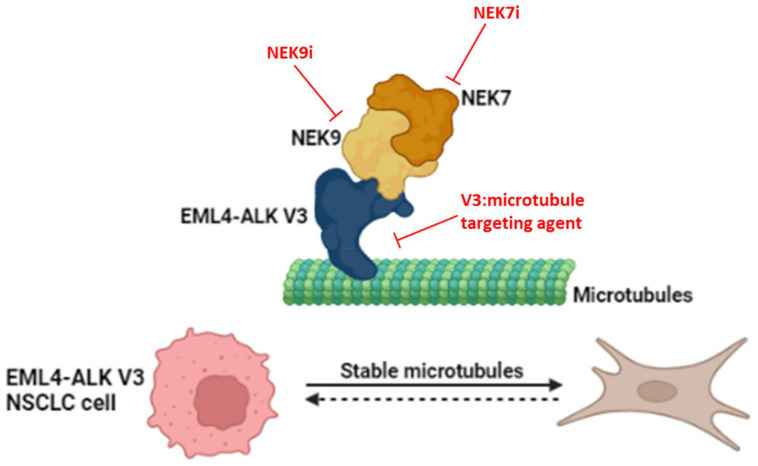
Potential therapeutic interventions based on the EML4-ALK V3/NEK9/NEK7 pathway. A cartoon illustrating how NEK9 is recruited to the microtubule network by EML4-ALK V3 and further recruits NEK7 forming the EML4-ALK V3/NEK9/NEK7 complex. The complex promotes increased cytoplasmic protrusions and enhanced migration in cells and potentially accelerated metastasis in patients. The use of targeted agents against the catalytic activities of NEK9 or NEK7, or that prevent assembly or microtubule recruitment of the complex, could potentially reverse these phenotypes.

It was further demonstrated that cells expressing EML4-ALK V3 recruit the NEK9 and NEK7 serine/threonine kinases, which are normally activated in mitosis, to interphase microtubules through binding to the NTD of EML4-ALK V3 [102]. Intriguingly, the expression of constitutively active mutants of NEK9 and NEK7 causes the same altered morphology and migration phenotypes in interphase cells lacking EML4-ALK proteins, while the depletion of NEK9 or NEK7 from cells expressing EML4-ALK V3 prevents induction of these phenotypes [102]. This provides persuasive evidence for an important signalling pathway activated by short EML4-ALK variants that is dependent on NEK9 and NEK7 rather than ALK catalytic activity. It also raises the prospect that inhibitors of NEK9 or NEK7 (or downstream substrates of these kinases) could be used as alternatives to ALK TKI monotherapy to potentially block the metastatic spread of tumours expressing these more aggressive variants (Figure 4).

Moreover, as EML4-ALK V3 recruits NEK7 and NEK9 to microtubules and promotes microtubule stabilization, it is plausible that microtubule association of the short EML4-ALK variants is required for activation of this pathway. If so, targeted agents that block microtubule association of these variants could also provide additional alternatives to ALK TKI monotherapy, although the rationale for this remains to be experimentally determined.

## 4. Combination of ALK Inhibitors and Chemotherapy

The large majority of cancer patients receive chemotherapy at some point during their treatment. The chemotherapeutic combination of platinum and pemetrexed was approved as first-line therapy for ALK+ NSCLC patients prior to the introduction of targeted ALK inhibitors but did not benefit all patients. However, the progression-free survival of ALK+ NSCLC patients treated with crizotinib was significantly higher (~8 months) than those treated with pemetrexed chemotherapy (~3 months) and a greater proportion of patients responded to crizotinib (~65%) than to chemotherapy (~20%) [43,103,104]. Interestingly, a retrospective analysis demonstrated that administration of platinum with pemetrexed in ALK+ NSCLC patients who had disease progression following ALK TKI therapy, prolongs PFS of these patients. Furthermore, when this chemotherapy was combined with an ALK inhibitor, higher PFS rates were observed as compared to chemotherapy alone, which suggests that the combination regimen may maintain sensitivity to ALK TKI therapy [105].

In a novel experimental approach, Li et al. (2018) used gold nanoparticles to deliver doxorubicin as well as reagents to simultaneously knock-down ALK to EML4-ALK V3 patient-derived cells. This system enables enhanced drug-loading capacity as well as tumour-specific targeting and led to a synergistic response both in vitro and in vivo [106].

EML4-ALK V3 not only associates with the microtubule network but also promotes microtubule stabilization, whereas V1 is found in the cytoplasm and does not obviously affect microtubule dynamics [29]. Microtubule poisons have been regularly used as chemotherapies for many tumour types, including NSCLC. One example is vincristine, a vinca alkaloid chemotherapy that promotes microtubule depolymerization by binding to tubulin monomers [107]. Sampson et al. (2022) sought to test the benefit of combining vincristine with ALK inhibitors in EML4-ALK patient-derived cells. They found that the combination of vincristine together with crizotinib or ceritinib led to an increased loss of cell viability and enhanced apoptosis in cells expressing V1, but not V3 [108]. This combination led to the inhibition of multiple ALK-dependent signalling pathways. Furthermore, they hypothesized that the resistance to vincristine observed in cells expressing V3 is due to the increased microtubule stability that results from binding of V3 to microtubules [108].

**Figure 5:**
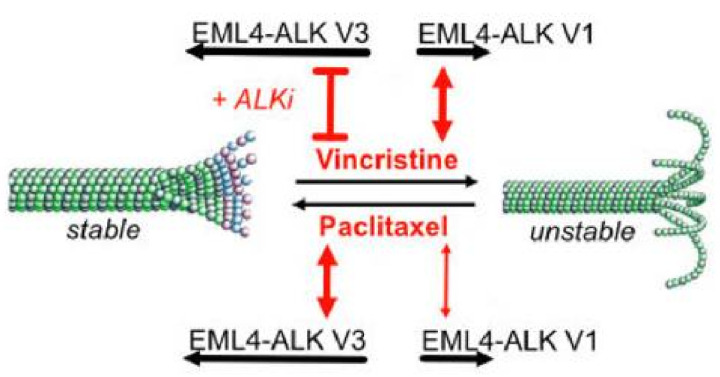
Combination of microtubule poisons and ALK inhibitors in EML4-ALK-driven NSCLC. Microtubules are dynamic structures and the balance between their polymerization and depolymerization determines microtubule stability. The microtubule poisons, vincristine and paclitaxel, promote the depolymerization and polymerization of microtubules, respectively. EML4-ALK V3 strongly stabilizes microtubules, whereas EML4-ALK V1 has a weak destabilizing effect. In combination with ALK TKIs, microtubule poisons that act in the same direction of microtubule stability could lead to enhanced loss of cell viability in EML4-ALK V1 and V3 patient tumours.

In a complementary study, Lucken et al. (2022) reported that paclitaxel, a microtubule stabilizing agent, gave a synergistic response in terms of loss of cell viability when combined with the ALK TKIs, alectinib and ceritinib, in cells expressing both V1 and V3. However, the response was greater in cells expressing V3, consistent with the increased microtubule stability that this protein causes. Furthermore, they found that the EML4-ALK V3 protein localised to the mitotic spindle microtubules with cells exhibiting stabilized K-fibres and increased mitotic defects including multipolar spindles, lagging chromosomes in metaphase and misaligned chromosomes in anaphase compared to cells expressing V1. Finally, the activity of the spindle assembly checkpoint was found to be reduced in cells expressing V3 compared to V1 [109]. Taken together, these two studies demonstrate the potential of combining ALK TKIs with microtubule poisons or targeted mitotic agents as an alternative to ALK inhibitor monotherapy (Figure 5). However, they again highlight the importance of identifying the specific EML4-ALK variant present when selecting such a combination treatment.

## 5. Combination of ALK Inhibitors and Radiotherapy

For patients with significant comorbidities, contraindications, or those who decline surgery, radiotherapy (RT) is a viable alternative, particularly with the introduction of stereotactic ablative radiotherapy (SABR) [110]. A combination of chemotherapy and RT is widely used in NSCLC patient treatments; however, the benefit of combining ALK TKIs with radiotherapy has not been thoroughly explored [111]. Despite its extensive use to treat ALK+ NSCLC, the first-generation ALK inhibitor, crizotinib, is not a selective ALK TKI and can inhibit other kinases such as cMET [112,113], which is a potential driver of RT resistance [114]. With that in mind, Dai et al. (2015) tested the effect of combining crizotinib with RT in patient-derived cells expressing V1. Encouragingly, the combination conferred a synergistic effect in reducing the proliferative capacity of these cancer cells and promoting apoptosis. Furthermore, they explored different modes of RT with crizotinib and suggested that crizotinib can in fact sensitize V1 cells to carbon ion RT to a greater extent than conventional RT [111]. Further evidence supports the potential of ALK TKIs to sensitize V1 cells to high linear energy transfer (LET) particle therapy with carbon ions. More specifically, a combination of TAE684 with carbon ion therapy resulted in a more profound reduction in the viability of V1 cells compared to either therapeutic approach alone [115].

A case study also reported a higher PFS in a patient diagnosed with EML4-ALK V3-driven NSCLC following treatment with stereotactic body radiotherapy (SBRT) plus alectinib compared to lorlatinib alone [116]. This indicates that RT can improve the sensitivity to distinct ALK TKIs and should be considered as an alternative to ALK TKI monotherapy given an acceptable safety profile.

Apart from determining suitable combination treatments, the order in which different therapies are administered must be considered. Antoni et al. (2020) used two different TKIs, crizotinib and PF-06463922 (a ROS inhibitor), together with RT to determine which therapy should be given first to allow maximal efficacy of the combination. They demonstrated that giving the TKIs after irradiating the tumour can increase the DNA damage in both EML4-ALK V1 and V3 patient-derived cells [117]. In fact, Sun et al. (2013) had previously demonstrated the ability of RT to sensitize V1 cells to crizotinib, observing enhanced apoptosis and growth inhibition of V1 cells both in vitro and in vivo when cells were treated with crizotinib following irradiation [118]. In addition, a case study reported a patient diagnosed with EML4-ALK V1-driven NSCLC who achieved a complete clinical response after being treated with chemotherapy plus RT, followed by crizotinib and alectinib [119]. In contrast, another study in which ALK TKIs were used prior to RT, found that the combination of ALK inhibitors with RT conferred similar results to RT alone, albeit superior to ALK TKI monotherapy [120]. Although these studies suggest a potential advantage in combining ALK inhibitors with RT, they also highlight the importance of the order in which therapies are administered as well as the consideration of toxicities and patient tolerability.

## 6. Combination of ALK Inhibitors and Immunotherapy

**Figure 6:**
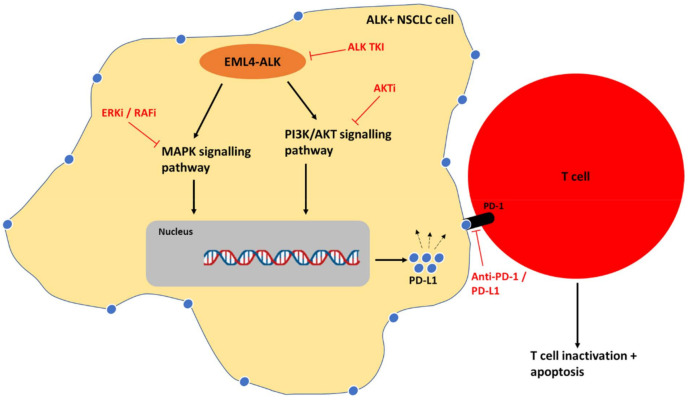
Combined EML4-ALK inhibition with PD-1/PD-L1-directed immunotherapy. This schematic figure illustrates how PD-L1 upregulation is regulated by EML4-ALK through the MAPK and PI3K/AKT signalling pathways. PD-L1 molecules on the surface of ALK+ NSCLC tumour cells interact with PD-1 receptors on T cells to promote their inactivation and apoptosis. A combination of ALK TKIs and PD-1/PD-L1 axis inhibition, as well as targeting the signalling pathways activated by ALK, could be effective approaches to promote activation of the immune system and tumour eradication.

The advent of immunotherapy has allowed for novel treatment avenues to be explored that encourage the immune system of a patient to promote tumour eradication [121]. Immune checkpoint inhibition is one of the most common types of immunotherapy and promotes activation of the immune system through the use of monoclonal antibodies that block the interaction between inhibitory molecules on the surface of tumour cells and their corresponding receptors on T cells or antigen presenting cells [121,122]. In fact, the upregulation of one such inhibitory ligand, programmed death ligand 1 (PD-L1), was observed on the surface of patient-derived cells expressing EML4-ALK V1, V3 and crizotinib-resistant V3 [123]. Interestingly, treatment with an ALK inhibitor led to a decrease in expression of PD-L1 in these cells. Hong et al. (2016) proposed that PD-L1 expression is regulated by EML4-ALK through the ERK and PI3K/AKT pathways, and that inhibiting these pathways could reverse its upregulation. In addition, PD-L1 on the surface of these tumour cells promoted T cell apoptosis through interaction with the corresponding receptor, PD-1, and inhibition of PD-1 with anti-PD-1 monoclonal antibodies not only prevented T cell apoptosis, but also promoted their activation. Finally, even though combination of PD-1 and ALK inhibition conferred similar results to either monotherapy alone, anti-PD-1 monoclonal antibodies can be considered as a possible additional treatment for EML4-ALK-driven NSCLC as well as an alternative for crizotinib-resistant patients (Figure 6) [123].

However, more research is needed to better understand when such combinations might be of use. For example, Pyo et al. (2020) demonstrated that anti-PD-1 therapy had no effect on murine models of ceritinib-resistant EML4-ALK, and that combination of anti-PD-1 monoclonal antibodies plus ceritinib conferred similar results to ceritinib monotherapy and was associated with severe side effects. It was observed that the level of intratumoral regulatory T cells was increased in ceritinib-resistant mice [124]. Regulatory T cells are an immunosuppressive subset of immune cells that can promote the inactivation of CD8+ effector T cells [125], which could explain the reduced efficacy of PD-1 checkpoint inhibitor therapy. Furthermore, clinical trials exploring the use of anti-PD-1/PD-L1 monoclonal antibodies in ALK+ NSCLC as monotherapies or in combination with ALK inhibitors have so far met with disappointing results or been terminated early following emergence of severe side effects [126,127,128].

Multiple studies have shown that the number of tumour-infiltrating cytotoxic T cells is reduced and their function impaired following PD-1/PD-L1 inhibition therapy [129,130]. Human leukocyte antigen class I (HLA I) is an essential molecule for antigen presentation to effector T cells [131], and Mu et al. (2022) found that expression of EML4-ALK V1 and V3 induces downregulation of HLA I molecules. This would explain the reduced number of activated CD8+ T cells in these tumours. They also found that treatment with ALK inhibitors could reverse HLA I downregulation through the MAPK pathway. Hence, a combination of ALK and MAPK inhibitors might enable maintained HLA I expression, increasing the efficacy of immunotherapies [132]. It will be interesting to assess whether HLA I expression is also altered in ALK TKI-resistant EML4-ALK cells, as this could uncover a novel targeting route for patients who have developed resistance.

Finally, cancer vaccination is an increasingly attractive immunological approach for prevention in those with a family history of cancer, or as an additional form of treatment through generation of anti-tumorigenic antibodies [133]. Codony-Servat et al. (2021) have explored the potential use of EGF immunization in EML4-ALK-driven NSCLC. Secretion of both EGF and TGFα from patient-derived cells expressing EML4-ALK V1 and V3 following ALK TKI treatment led to resistance to ALK inhibitors, and this could be reversed with anti-EGF antibodies generated through vaccination. Not only was the efficacy of ALK inhibitors increased, but the proliferative capacity of the cancer cells was decreased whilst the apoptotic rate was increased. The combination of these vaccines with ALK TKIs prevented activation of the EGFR bypass pathway and delayed the emergence of resistance to ALK TKIs, which suggests the potential use of this combination to prevent or overcome resistance to ALK TKI monotherapy [134].

## 7. Conclusions

ALK inhibitor monotherapy is the standard of care treatment for patients with EML4-ALK-driven NSCLC. However, even with the generation of more efficacious ALK inhibitors, most patients inevitably relapse, highlighting the need for novel and alternative treatments. An incomplete understanding of the mechanisms underpinning EML4-ALK-driven cancer progression and how resistance develops presents a challenge to the search for alternative treatments and emphasizes the need for further research. In this review, we have summarized efforts to identify alternative treatment options to ALK TKI monotherapy that overcome intrinsic or acquired resistance in ALK+ NSCLC patients. This knowledge may be beneficial not only for patients with EML4-ALK fusions but also those with less common fusions of ALK to other partner proteins.

The use of targeted agents against pathways that are either dependent or independent of ALK activation, in combination with ALK TKIs, could prevent the emergence of resistance through the activation of bypass pathways or downstream effector molecules. Given that some EML4-ALK variants can bind and alter microtubule dynamics, it might be possible to use microtubule poison chemotherapies in combination with ALK TKIs to interfere with mitotic progression and promote cancer regression through the activation of apoptotic pathways, whilst inhibiting proliferative and survival pathways. There is a lot of debate about whether the combination of ALK inhibition and RT can be effective in preventing resistance. Nonetheless, most in vitro studies agree that RT can, on some level, enhance the anti-tumorigenic properties of ALK inhibitors; thus, their combination with RT should be further explored. Finally, immunotherapy represents an important avenue to pursue, either alone or in combination with ALK TKIs. Although some types of immunotherapy have proven to be ineffective so far (e.g., PD-1/PD-L1 inhibition), more research is required into other approaches, such as immunization.

This review highlights the significant amount of pre-clinical data that suggests that identification of the specific EML4-ALK variant each patient possesses would be helpful in predicting response to ALK inhibitor therapy, as distinct variants give dramatically different responses to both ALK TKI monotherapy and combination treatments. Hence, introduction of variant identification into routine clinical practice will enable more informed decisions to be made on treatment selection. However, despite our growing understanding of the resistance mechanisms to ALK inhibitors in cultured cells, there remains insufficient data regarding their contribution to resistance in patients. Therefore, we propose that future research should focus on elucidating the importance of these and other resistance mechanisms in the clinic, further guiding selection of the most effective medicines for these patients.
